# Weight Stigma Reduction and Genetic Determinism

**DOI:** 10.1371/journal.pone.0162993

**Published:** 2016-09-15

**Authors:** Anja Hilbert

**Affiliations:** Integrated Research and Treatment Center Adiposity Diseases, Department of Medical Psychology and Medical Sociology, University of Leipzig Medical Center, Leipzig, Germany; McMaster University, CANADA

## Abstract

One major approach to weight stigma reduction consists of decreasing beliefs about the personal controllability of—and responsibility for—obesity by educating about its biogenetic causes. Evidence on the efficacy of this approach is mixed, and it remains unclear whether this would create a deterministic view, potentially leading to detrimental side-effects. Two independent studies from Germany using randomized designs with delayed-intervention control groups served to (1) develop and pilot a brief, interactive stigma reduction intervention to educate N = 128 university students on gene × environment interactions in the etiology of obesity; and to (2) evaluate this intervention in the general population (N = 128) and determine mechanisms of change. The results showed (1) decreased weight stigma and controllability beliefs two weeks post-intervention in a student sample; and (2) decreased internal attributions and increased genetic attributions, knowledge, and deterministic beliefs four weeks post-intervention in a population sample. Lower weight stigma was longitudinally predicted by a decrease in controllability beliefs and an increase in the belief in genetic determinism, especially in women. The results underline the usefulness of a brief, interactive intervention promoting an interactionist view of obesity to reduce weight stigma, at least in the short term, lending support to the mechanisms of change derived from attribution theory. The increase in genetic determinism that occurred despite the intervention’s gene × environment focus had no detrimental side-effect on weight stigma, but instead contributed to its reduction. Further research is warranted on the effects of how biogenetic causal information influences weight management behavior of individuals with obesity.

## Introduction

People with obesity are pervasively stigmatized and exposed to weight-related discrimination in many domains of life [1]. Posing a threat for mental and physical health and well-being [2–4], interventions to reduce weight stigma are being developed. One major approach consists of decreasing beliefs about the personal controllability of body weight by educating about the biogenetic causes of obesity; however, this has been inconsistently efficacious [5]. In addition, it remains unclear whether focusing on biogenetic causes promotes a deterministic view of individuals with obesity as being genetically and thus, fundamentally and inevitably different [6], potentially exacerbating stigma as a side-effect.

Stigmatizing attitudes towards individuals with obesity emerge in the context of beliefs about the controllability of and responsibility for the excess body weight [7–10]. As predicted by the prominent attribution theory [11], the more a stigma such as weight stigma is attributed to internal, controllable causes, and, thus, responsibility is perceived, the greater are one’s negative reactions to it, including, for example, negative emotions, stigmatizing attitudes, and behavioral discrimination. In line with these predictions, attributions to uncontrollable causes, such as biogenetic causes, have been shown to co-occur with lower stigmatizing attitudes in most [12,14,16,17], but not all studies on weight stigma [13,15].

Providing information on the biogenetic causes of obesity has shown mixed effects on stigmatizing attitudes [18]. Some experimental studies that briefly informed adult participants in student and community-based samples about the biogenetic causes of obesity found reduced stigmatizing attitudes [7,19–22], while others did not [13,17,23,24]. Interpreting results is complicated by methodological shortcomings (e.g., lack of adequate control groups, no pre-post design), so that causality in some studies cannot clearly be determined. In contrast to these brief experimental manipulations, more comprehensive, multi-component interventions that were designed to reduce stigmatizing attitudes by using education on genetic factors, among other interventions (e.g., information on the sociocultural background), led to decreased explicit stigmatizing attitudes over several weeks of follow-up in students [25–29]. One comprehensive, multi-component intervention in a student sample even reduced implicit, unconscious stigmatizing attitudes [27], as assessed by the Implicit Association Test (IAT) [30]. In contrast, an anti-stigma film [28] and a brief experimental manipulation of biogenetic causes [24] did not reduce implicit stigmatizing attitudes in students.

Despite the increasing evidence base on the stigma-reducing potential of providing information on the biogenetic causes of obesity, little research has been conducted on the potential mechanisms and side-effects of interventions [31]. Some studies documented a specific decrease in controllability beliefs [21,25,27,28], as would be predicted by attribution theory [11]. It has not been consistently demonstrated whether interventions increased biogenetic causal attributions [23,27,29], or had no impact on these attributions [13,20]. It further remains unclear whether internal causal attributions to an individual’s behavior decreased, whether knowledge of obesity and its etiology increased, and whether the belief of obesity to be genetically determined, involving the view of people with obesity as fundamentally different, was heightened.

While most of the aforementioned experimental manipulations presented an essentialist, deterministic view of obesity according to which genes form the basis of individuals and determine his/her behavior or body weight, thereby limiting personal control and responsibility, multi-component studies sought to provide a more complex, multi-factorial view of obesity in order to reduce weight stigma. For any provision of genetic causal information, it has been argued that the reference to genetic factors could be misinterpreted in an essentialist way: Genetic essentialism [32], because of its potential to promote perceptions of fundamental genetic differentness, could lead to increased stigma as a side-effect [6,31,33]. Indeed, a meta-analysis on the stigma of mental illness found that a stronger consideration of biogenetic causes was associated with less blame, but views of mental illness as more dangerous [34]. While in the obesity field none of the aforementioned experimental or multi-component stigma reduction studies documented increased weight stigma after providing genetic causal information, Persky and Eccleston [20] found that information on the genetic causes of obesity was associated with a reduced readiness of medical students to recommend weight loss, exercise, and diet to patients with obesity in a virtual treatment setting. Thus, more research is warranted on potential mechanisms and side-effects, including causal attributions, genetic determinism, and knowledge.

Two independent studies were designed (1) to develop and pilot a brief, interactive multi-component intervention on weight stigma reduction with a focus on providing a gene × environment model of obesity etiology in a student sample; and (2) to evaluate effects of this intervention in the general population over a prolonged follow-up. It was hypothesized that the intervention would reduce explicit stigmatizing attitudes (primary outcome) and controllability beliefs; leave implicit stigmatizing attitudes unchanged—based on the aforementioned inconsistent findings on the variability of implicit stigmatizing attitudes; and increase knowledge, genetic causal attributions, and genetic determinism, but decrease internal causal attributions (secondary outcomes).

## Study 1: Development of a Brief, Interactive Intervention for Weight Stigma Reduction Providing Gene × Environment Causal Information

### Materials and Methods

#### Participants and procedure

Participants were 128 university students (79 women, 49 men) recruited for the study at Philipps University of Marburg, Germany. Participants were on average 23.01 ± 3.24 years old and had a mean body mass index (BMI, kg/m^2^) of 22.41 ± 3.00 kg/m^2^ that was calculated based on self-reported height and weight. Using the guidelines by the World Health Organization [35], overweight or obesity were identified in 21 (16.4%) participants (BMI ≥ 25.00 kg/m^2^).

Of the 128 participants, 64 individuals were randomly assigned to the experimental group to receive the intervention, and 64 individuals were assigned to a delayed-intervention control group that was offered the intervention after the post-intervention assessment. A delayed-intervention control group is an appropriate control condition in the early stage of intervention development. The two groups did not differ significantly in the distribution of gender, [χ^2^ (1, N = 128) = 1.62], age [F(1, 126) = 0.45], BMI [F(1, 126) = 3.34], or weight status [underweight/normal weight vs. overweight/obese; χ^2^(1, N = 128) = 2.79; all p > .05].

Participants were seen individually in the laboratory suite. Prior to all study procedures, they were informed about the study in a verbal and written format, and written informed consent was obtained. Consent forms and personal information collected therein were archived at the University in a locked facility and locked cabinet, so that only study personnel had access to them. When the study was conducted from 01/2006-04/2007, it was not mandatory to seek approval by an Institutional Review Board for studies in non-clinical participants in Germany. There was no Institutional Review Board in place for non-clinical studies from the Psychology Department of Philipps University of Marburg at that time.

For the baseline assessment, participants completed the measures described below. Participants of the experimental group then received the computerized intervention. Participants of both groups were given an appointment for the post-intervention assessment 10 to 16 days after the initial appointment in order to complete the same measures as at baseline. Participants of the control group were offered the intervention at this time point. All participants were offered two hours of research participation credits as incentive.

#### Measures

The Antifat Attitudes Test (AFAT) [19] was used to assess the primary outcome of explicit stigmatizing attitudes. The AFAT includes 47 statements to be rated on a 5-point Likert rating scale ranging from 1 = *Definitely disagree* to 5 = *Definitely agree* (e.g., “Fat people have no will power”). A total mean score was calculated, with higher scores indicating greater explicit stigmatizing attitudes. The German version, controlled by a retranslation procedure, demonstrated an excellent internal consistency of the total mean score (Cronbach α’s = .96) in this study’s sample that was comparable to the original AFAT’s internal consistency [19].

Regarding secondary outcomes, the Beliefs About Obese Persons Scale (BAOP) [36] was used to measure explicit beliefs about diverse personal and biological causes of obesity and the controllability of body weight of individuals with obesity. Participants are asked to rate eight statements about the causes of obesity on a 6-point Likert scale ranging from -3 = *I strongly disagree* to +3 = *I strongly agree* (e.g., “Obesity is usually caused by overeating”). Higher sum scores (with a maximum value of 24) indicate a stronger belief that obesity is not under personal control. The German translation of the BAOP was controlled by a retranslation procedure. Internal consistency of the BAOP in this study’s sample was adequate (Cronbach α = .77) and comparable to that reported for the original version [36].

Further, a test of knowledge was constructed to measure the participants’ knowledge about the etiology of obesity, weight stigma, and modifiability of body weight through weight loss interventions. This test included 10 items offering four response options each, one of which was correct (e.g., “In family and twin studies, how much is body weight influenced by genetic factors?” 10–20%, 20–50%, 30–70% [correct response], 70–100%). Correct answers were summed up, with a possible range of 0 = *No knowledge about obesity* to 10 = *Very knowledgeable about obesity*.

To measure implicit stigmatizing attitudes, a computerized version of the IAT [30] was used to determine the relative strength of associations between a pair of opposing attribute and target categories. In weight stigma IATs, probands classify target stimuli (e.g., skinny, plump) into a thin or fat category, and attribute stimuli (e.g., smart, stupid) into a positive or negative category. Responses are typically more correct or faster for compatible (e.g., fat and negative) than for incompatible (e.g., fat and positive) pairings [37,38]. The weight stigma IAT in this study used culturally adapted items from Teachman and Brownell [38] and followed the structure recommended by Nosek, Greenwald, and Banaji [39]. As the dependent variable, the D score was calculated by dividing the mean differences of reaction times by standard deviations of all reaction times of the trials presenting compatible and incompatible anti-fat pairings [40]. Higher D scores are indicative of a stronger association between fat and negative, and thus of stronger implicit stigmatizing attitudes.

#### Intervention

The stigma reduction intervention sought to make participants reflect about their view on the controllability and responsibility of individuals with obesity regarding their excess weight. The intervention was delivered through an interactive audio-visual slide show (55 slides), presented via Microsoft PowerPoint (Microsoft Corporation, Redmond, Washington State, USA), and took about 60 minutes to complete. In order to involve participants in the reflection about their own thoughts and attitudes about overweight people, the intervention used psychoeducation, guided discovery, and mental imagery techniques. Additional material was provided that the participants were asked to work through during the intervention (e.g., comparison of current and desired body size).

To introduce the topic, participants were asked in a guided imagery task to imagine encountering an overweight woman in a public place, and were instructed to observe their reactions using a behavior analytic framework of the emotional, cognitive, behavioral, and perceptual levels. In Module 1, participants received general information on the definition, prevalence, risk factors, and treatment of obesity. The emphasis was on the etiological relevance of genetic factors in interaction with environmental factors and on the limited modifiability of body weight through weight loss treatment. Module 2 addressed weight stigma in the context of the current societal pressure to be slim. Using body image interventions [41], relativity of the beauty ideal was addressed by showing paintings of women at different sizes from different eras. In addition, participants were made aware of their own beauty ideal and current body size using Stunkard’s Standard Figural Stimuli [42]. Module 3 addressed prejudice against people with obesity. Current research was presented on the origin of weight stigma, forms of weight-related stigmatization and discrimination, and the consequences for people with obesity. A reflection task was designed to make participants aware of their own prejudice against individuals with obesity. The presentation was concluded by summarizing take-home messages.

#### Data analytic plan

Post-intervention group differences on the primary and secondary outcome variables were analyzed using univariate analyses of variance with baseline values as covariates (ANCOVAs). Change over time was analyzed by conducting repeated measures ANOVAs of Group (experimental, control group; between-subjects) × Time (baseline, post-intervention; within-subjects). In order to determine influences by participants’ body weight, weight status (underweight/normal weight vs. overweight/obesity) was entered as a covariate in an additional step in the analyses outlined above; the results were reported only if changed. Partial η^2^, describing the proportion of total variability attributable to a factor, was displayed to estimate effect size (η^2^, small: ≥ 0.01, medium: ≥ 0.06, large: ≥ 0.14) [43]. A two-tailed α of .05 was applied to all tests. Analyses were performed using SPSS 22.0 (SPSS Inc., Chicago, Illinois, USA). The sample size of N = 128 provided 80% power to detect a medium effect size for a Group × Treatment effect of η^2^ = 0.06. Data are made available in the Supporting Information (S1 Data).

### Results

As summarized in Table 1, post-intervention ANCOVAs showed significantly less explicit stigmatizing attitudes (AFAT) in the experimental than in the control group (p < .01; medium effect). In addition, less controllability beliefs (BAOP), and greater knowledge of obesity were found (both p < .001; large effects). Implicit stigmatizing attitudes (IAT) did not differ between groups (p > .05).

**Table 1:** Study 1: Effects of a brief interactive stigma reduction intervention over two weeks in a student sample (N = 128).

	Experimental group				Control group									
	Baseline		Post-intervention		Baseline		Post-intervention		Group^a^		Group x Time^b^		Time^b^	
	M	SD	M	SD	M	SD	M	SD	F(1, 125)	η^2^	F(1, 126)	η^2^	F(1, 126)	η^2^
**Explicit measures**														
Stigmatizing attitudes (AFAT)	1.98	0.47	1.85	0.41	2.25	0.65	2.22	0.69	7.36**	0.06	4.23*	0.03	9.42***	0.07
Controllability beliefs (BAOP)	18.00	7.70	25.27	6.66	16.52	6.85	18.30	7.47	36.06***	0.22	22.76***	0.15	61.94***	0.33
Knowledge of obesity	3.70	1.58	5.80	1.90	3.53	1.41	3.78	1.33	50.72***	0.29	33.48***	0.21	54.10***	0.30
**Implicit measure**														
Stigmatizing attitudes (IAT-D)	0.56	0.37	0.57	0.43	0.63	0.38	0.63	0.42	0.05	0.00	0.01	0.00	0.09	0.00

AFAT indicates Antifat Attitudes Test (1–5); BAOP, Beliefs About Obese Persons Scale (0–48); Knowledge of obesity (0–10); IAT, Implicit Association Test, D, mean differences of reaction times divided by standard deviations of all reaction times when presenting compatible and incompatible anti-fat pairings.

^a^Univariate analysis of variance by Group (EG, CG; between-subjects) with baseline values as covariates.

^b^Repeated measures analysis of variance of Group (EG, CG; between-subjects) × Time (baseline, post-intervention; within-subjects).

*p < .05

**p < .01

***p < .001

Repeated measures ANOVAs revealed significant Group × Time effects for explicit stigmatizing attitudes (p < .05; small effect), and for controllability beliefs and knowledge of obesity (both p < .001; medium-to-large effects). Post-hoc analyses showed that explicit stigmatizing attitudes decreased significantly and knowledge increased significantly in the experimental group (both p < .01; η^2^ = 0.11, 0.47), while in the control group both variables remained unchanged (both p > .05; η^2^ = 0.03, 0.04). In addition, both groups showed a large-size decrease in controllability beliefs post-intervention when compared to baseline, but the effect size was more than twice as high in the experimental group than in the control group (all p < .05; η^2^ = 0.44 vs. 0.17). Implicit stigmatizing attitudes did not show any significant effect in the Group × Time analysis (both p > .05).

### Discussion

The results demonstrated that the brief interactive intervention for weight stigma reduction yielded a significant but small reduction in explicit stigmatizing attitudes two weeks after the intervention. In addition, the intervention led to medium-to-large decreases in controllability beliefs and increases in knowledge, as predicted. In contrast, the control group showed unchanged scores in knowledge and slightly decreased scores in controllability beliefs, which may result from participating in the study, possibly stimulating a reflection about attitudes towards people with obesity. Overall, the results confirm previous evidence on the destigmatizing potential of multi-component programs [25–29], even with the brief time commitment of the intervention (60 minutes).

In contrast to the significant effects on explicit measures, no effects emerged for implicit stigmatizing attitudes, as expected, which reflects some previous research on relatively brief interventions [24,28], but is not consistent with the results from a more comprehensive health curriculum [27]. Although the interactive intervention was designed to activate implicit stigmatizing attitudes (e.g., by guided imagery), the intervention may not have been potent enough to substantially change them.

Based on these results, further evidence was deemed necessary to determine whether this intervention would: be useful in a population-based sample; show maintenance of effects over a prolonged follow-up; modify causal attributions and increase genetic knowledge of obesity; and increase the belief in genetic determinism even if it was based on a gene × environment model.

## Study 2: Effects of a Brief, Interactive Weight Stigma Reduction Intervention on Stigmatizing Attitudes and Genetic Knowledge, Attributions, and Determinism

### Materials and Methods

#### Participants and procedure

A total of 128 individuals (79 women, 49 men) were recruited from the community for the study in the region of Marburg, Germany, using flyers, public notices, and newspaper announcements. Participants were on average 35.31 ± 12.54 years old and had a mean BMI of 25.64 ± 5.64 kg/m^2^ (calculated based on self-reported height and weight), with overweight or obesity identified in 54 (42.2%) participants (BMI ≥ 25.00 kg/m^2^). Of the 128 participants, 63 individuals were randomly assigned to the experimental group to receive the intervention, and 65 individuals were assigned to a delayed-intervention control group. The two groups did not differ significantly in the distribution of gender [χ^2^(1, N = 128) = 1.10], age [F(1, 126) = 1.27], BMI [F(1, 126) = 0.04], or weight status [underweight/normal weight vs. overweight/obesity; χ^2^(1, N = 128) = 0.02; all p > .05]. However, significantly more participants in the experimental group than in the control group had a lower level of education [< 13 years vs. ≥ 13 years of school education; experimental group: 39, 61.9%, control group: 26, 40.0%; χ^2^(1, N = 128) = 6.14, p < .05].

The procedure, including informed consent, followed that in Study 1. As opposed to Study 1, participants were seen four weeks instead of two weeks after baseline for their post-intervention assessment. The study was conducted from 09/2008-12/2009. Participants were offered 25 EUR as an incentive.

#### Measures

As in Study 1, the AFAT was used as the primary outcome measure, and the BAOP and the computerized IAT were used as secondary outcome measures to assess explicit stigmatizing attitudes, controllability beliefs, and implicit stigmatizing attitudes. The following additional secondary outcome measures were assessed.

A test on the genetic knowledge of obesity was created in order to evaluate interventional effects on the specific knowledge about genetic factors of obesity. Eight items offered three to five response options, one of which was correct (e.g., “Only maternal genes predispose for the development of obesity.” This is right, this is wrong [correct response], I don’t know). Correct answers were summed with a range of 0 = *No knowledge about obesity genetics* to 8 = *Very knowledgeable about obesity genetics*.

To assess causal attributions of obesity, the 11-item Causal Attributions of Obesity Questionnaire (CAOQ) was used [12]. This questionnaire includes three subscales on the perceived risk factors of obesity: Behavior (or internal attributions), which encompassed eating and activity behavior likely leading to a positive energy balance (e.g., “Binge eating,” “Lack of physical activity;” 5 items); Environment (or external attributions), which referred to the obesogenic food and activity environment (e.g., “Healthy food is too expensive,” “Lack of facilities for outside physical activity;” 5 items); and Heredity (genetic attributions; “Obesity is something that is inherited from the parents;” 1 item). All scales were derived from principal components analysis [44]. Items were given five-point rating scales (1 = *Disagree completely* to 5 = *Agree completely*) and subscale means were used for the analysis.

An adapted version of the Belief in Genetic Determinism Scale (BGDS) [45] was used for this study to measure how much participants believed that obesity is determined by genetics. Of the original 18 items, 10 items with the highest explanation of variance of the total mean score were selected for this study and adapted to obesity (e.g., “The fate of each person lies in his or her genes.”). Items, averaged to a mean score, were supplied with 7-point rating scales ranging from 1 = *Not at all true* to 7 = *Completely true*. The adapted scale revealed an adequate internal consistency in this study’s sample (Cronbach α’s = .73), comparable to that reported for the original version [45].

#### Control variable

The Social Desirability Scale-17 (SDS-17) [46] was used to control for the effect of social desirability in how participants answered in the outcome measures. The SDS-17 consists of 17 items to be answered with *right* or *wrong*. To analyze, socially desirable answers were summed (e.g., “During arguments I always stay objective and matter-of-fact”). Stöber [46] reported adequate retest-reliability (*r*_*tt*_ = .82).

#### Intervention

The intervention developed and piloted in Study 1 was applied, with minor modifications (e.g., reduced difficulty level of scientific content).

#### Data analytic plan

The same data analytic strategy as in Study 1 was used, with three adaptations: (1) The dependent variables included causal attributions subscale scores, genetic knowledge of obesity sum score, BGDS total score, beyond AFAT total score, BAOP sum score, and IAT-D score. (2) Education was used as a covariate in the analyses. (3) In order to determine effects of participants’ social desirability, the SDS-17 sum score was entered as a covariate in an additional step in the analyses; the results were reported only if changed. In addition to these analyses, a stepwise multiple linear regression analysis was conducted, controlling for sociodemographic variables in step 1, in order to identify predictors of explicit stigmatizing attitudes (effect size evaluation: small: R^2^ ≥ .02, medium: ≥ .15, large: ≥ .35). To select predictors, zero-order associations with post-intervention AFAT total score were determined using Pearson's r or Spearman's rho correlation coefficients for the following continuous or ordinal variables: group status, gender, age, weight status, education, baseline AFAT total score, and baseline and change scores (post-intervention minus baseline) of all secondary outcome variables. The variables showing at least small-effect associations with the post-intervention AFAT total score were retained for regression analysis (small: r ≥ .10, medium: ≥ .30, large: ≥ 0.50). A two-tailed α of .05 was applied to all tests. Analyses were performed using SPSS 22.0 (SPSS Inc., Chicago, Illinois, USA). Data are made available in the Supporting Information (S2 Data).

### Results

#### Change by group and time

As summarized in Table 2, ANCOVAs did not show any group difference in post-intervention explicit stigmatizing attitudes (AFAT), controlling for baseline values and education (p > .05). Likewise, there were no significant effects for controllability beliefs (BAOP), internal attributions (CAOQ), or implicit stigmatizing attitudes (IAT; all p > .05). In contrast, genetic knowledge of obesity, genetic causal attributions (CAOQ), and belief in genetic determinism (BGDS) were greater in the experimental group than in the control group (medium to large effects; p < .01).

**Table 2:** Study 2: Effects of a brief interactive stigma reduction intervention over four weeks in a general population sample (N = 128).

	Experimental group				Control group									
	Baseline		Post-intervention		Baseline		Post-intervention		Group^a^		Group x Time^b^		Time^b^	
	M	SD	M	SD	M	SD	M	SD	F(1, 125)	η^2^	F(1, 125)	η^2^	F(1, 125)	η^2^
**Explicit measures**														
Stigmatizing attitudes (AFAT)	2.10	0.53	2.15	0.50	2.08	0.51	2.12	0.49	0.00	0.00	0.01	0.00	1.92	0.02
Controllability beliefs (BAOP)	18.42	7.92	20.44	7.51	17.64	5.93	19.06	7.05	0.71	0.01	0.29	0.00	0.81	0.01
Genetic knowledge of obesity	2.40	1.53	3.54	1.54	2.52	1.50	2.54	1.56	28.37***	0.19	23.72***	0.16	0.13	0.00
Genetic attributions (CAOQ)	2.92	0.89	3.41	0.94	2.85	1.00	2.89	1.03	10.20**	0.08	6.07*	0.05	0.27	0.00
Internal attributions (CAOQ)	3.98	0.56	3.79	0.74	3.99	0.49	3.87	0.53	0.09	0.00	0.06	0.00	7.51**	0.06
External attributions (CAOQ)	3.16	0.64	3.06	0.60	3.21	0.60	3.12	0.64	0.00	0.00	0.02	0.00	2.80	0.02
Genetic determinism (BGDS)	4.10	0.73	4.67	0.90	4.05	0.88	4.15	0.91	13.33***	0.10	10.97**	0.08	3.78	0.03
**Implicit measure**														
Stigmatizing attitudes (IAT-D)	0.51	0.31	0.45	0.25	0.47	0.32	0.41	0.34	0.06	0.00	0.00	0.00	0.48	0.00

AFAT indicates Antifat Attitudes Test (1–5); BAOP, Beliefs About Obese Persons Scale (0–48); Genetic Knowledge of Obesity (0–8); CAOQ, Causal Attributions of Obesity Questionnaire (1–5); BGDS, Belief in Genetic Determinism Scale (1–7); IAT, Implicit Association Test, D, mean differences of reaction times divided by standard deviations of all reaction times when presenting compatible and incompatible anti-fat pairings.

^a^Univariate analysis of variance by Group (EG, CG; between-subjects) with baseline values and education as covariates.

^b^Repeated measures analysis of variance of Group (EG, CG; between-subjects) × Time (baseline, post-intervention; within-subjects) with education as covariate.

*p < .05

**p < .01

***p < .001

Repeated measures ANCOVAs did not reveal any significant effect on explicit stigmatizing attitudes, controllability beliefs, or implicit stigmatizing attitudes (all p > .05; Table 2). However, significant Group × Time effects for genetic knowledge of obesity, genetic causal attributions, and belief in genetic determinism emerged (small to large effects; all p < .05), but not on internal attributions (p > .05). Post-hoc analyses showed that genetic knowledge about obesity, genetic attributions, and the belief in genetic determinism increased significantly and at large effect sizes in the experimental group (all p < .001; η^2^ = 0.17, 0.37, 0.32), while in the control group these variables remained unchanged (all p > .05; η^2^ = 0.00, 0.00, 0.02). In addition, a significant main effect of time was found for internal attributions that decreased across both study groups (p < .05; medium effect). This effect was no longer significant when weight status or social desirability were included in the model as additional covariates (p > .05).

#### Prediction of post-intervention explicit stigmatizing attitudes

To select predictor variables in the multiple linear regression analysis, correlational analyses showed that lower post-intervention explicit stigmatizing attitudes had at least small-size correlations with: lower baseline explicit stigmatizing attitudes (r = .86) and controllability beliefs (-.35), and more genetic causal attributions, less internal and external causal attributions, and greater belief in genetic determinism (-.28 ≤ r ≤ .36). In addition, post-intervention explicit stigmatizing attitudes were lower the greater the increase in genetic determinism beliefs and the greater the decrease in controllability beliefs (-.19 ≤ r ≤ -.11). From the sociodemographic variables, female gender, higher age, and overweight/obesity revealed small-size associations with lower post-intervention explicit stigmatizing attitudes (-.11 ≤ r ≤ .19).

When entering these variables into a stepwise multiple linear regression analysis (Table 3), lower baseline stigmatizing attitudes (large effect), greater decrease in controllability beliefs, greater increase in genetic determinism beliefs, and female gender (small effects) showed significant prediction effects for lower post-intervention explicit stigmatizing attitudes (all p < .05). The final model including these predictors was significant [F(4, 123) = 113.96, p < .001] and explained 78% of the variance.

**Table 3:** Study 2: Multiple linear regression analysis: Prediction of explicit stigmatizing attitudes towards obesity (N = 128).

	B	SE	Beta	t test	R^2^	R^2^ change
Gender	0.09	0.04	0.09	2.13*	.03	.03
Baseline explicit stigmatizing attitudes (AFAT)	0.81	0.04	0.85	20.30***	.74	.71
Change controllability beliefs (BAOP)	-0.01	0.00	-0.16	-3.74***	.77	.03
Change genetic determinism (BGDS)	-0.07	0.03	-0.11	-2.51*	.78	.01
Constant	0.37	0.10				

Outcome variable: Stigmatizing attitudes towards obesity 4 weeks post-intervention (AFAT, Antifat Attitudes Test, 1–5). B indicates unstandardized regression coefficient; SE, standard error; Beta, standardized regression coefficient; R^2^, adjusted multiple R^2^ (cumulative); R^2^ change, adjusted multiple R^2^ (by predictor). Gender (female = 1, male = 2); BAOP, Beliefs About Obese Persons Scale (0–48); BGDS, Belief in Genetic Determinism Scale (1–7). Excluded predictor variables: age, baseline: controllability beliefs (BAOP); genetic, internal, and external causal attributions (CAOQ); genetic determinism (BGDS).

*p < .05

**p < .01

***p < .001

#### Additional analyses

Because of the differences between the experimental and the control group in educational level, additional analyses stratified by educational level were conducted (< 13 years or ≥ 13 years of school education). In the ANOVAs of Group (experimental, control group; between-subjects) × Time (baseline, post-intervention; within-subjects), in participants with high educational level only, controllability beliefs increased [F(1, 61) = 4.27, p < .05, η^2^ = 0.07] and implicit stigmatizing attitudes decreased over time [F (1, 61) = 4.70, p < .05, η^2^ = 0.07] in both the experimental and the control group, while genetic causal attributions increased over time specifically in the experimental group [F(1, 61) = 4.00, p < .01, η^2^ = 0.12]. In contrast, internal causal attributions decreased over time in participants with low educational level only [F(1, 63) = 10.82, p < .01, η^2^ = 0.15]. As reported for the total sample (see above), in both participants with high and low educational level, genetic knowledge and the belief in genetic determinism increased specifically in the experimental group (all p < .05).

In the regression analysis of participants with high educational level, lower baseline explicit stigmatizing attitudes (0.83), greater decrease in controllability beliefs (-0.16), greater increase in genetic determinism beliefs (-0.17), and greater genetic causal attributions (-0.13) significantly predicted lower post-intervention explicit stigmatizing attitudes (all p < .05). The final model including these predictors was significant [F(4, 62) = 67.86, p < .001] and explained 81% of the variance. In participants with low educational level, only lower baseline stigmatizing attitudes (0.85) and greater decrease in controllability beliefs (-0.18) were significant predictors (all p < .05). The final model including these predictors was significant [F(2, 64) = 96.62, p < .001] and explained 75% of the variance.

### Discussion

As opposed to Study 1, the brief interactive intervention to reduce weight stigma did not show any significant effect on explicit stigmatizing attitudes in a general population sample over a prolonged follow-up of four weeks. Neither were beliefs about the personal controllability of one’s overweight attenuated. Stratification of the analyses by educational level, however, showed a decrease in controllability beliefs and implicit stigmatizing attitudes in participants with higher educational level only, which applied to both the experimental and the control group. These results are consistent with meta-analytical results suggesting almost zero effects for weight stigma interventions that were conducted in individuals from the general population, while interventions in higher educated student samples tended to yield greater reductions in stigmatizing attitudes [18]. Beyond the population-based sample, the extended time frame of four weeks in this study may have attenuated effects that were found to be small after two weeks in Study 1. Further, older age was significantly associated with lower stigmatizing attitudes, so that age differences between studies are unlikely to be accountable. Overall, the results in this study were stable when weight status or social desirability were controlled.

Regarding mechanisms of change, the brief interactive intervention increased specific knowledge on obesity genetics and fostered genetic causal attributions as hypothesized, with the latter especially pronounced in individuals with high educational level. In contrast, unexpectedly, causal attributions to internal factors (e.g., overeating, lack of physical activity) decreased in both study groups, thus indicating that this reduction was not related to the intervention, but presumably to participating in the study provoking a reflection about the responsibility of the onset of obesity. This effect was not stable when controlled for covariates, and especially applied to individuals with low educational level. As hypothesized, the belief in genetic determinism was increased in the intervention group only, although the program’s focus was on gene × environment interactions. Thus, the reference to gene × environment interactions may have been misunderstood in a deterministic way. However, four weeks after the intervention, the participants still scored—with low variability—close to the BGDS mean (M = 4.67, SD = 0.90 vs. scale M = 4); thus, they were unlikely to perceive obesity as fully genetically determined. Unlike preliminary results on potential negative side-effects of genetic causal information on students’ weight loss recommendations or overeating behavior [16,20], deterministic beliefs were not shown to have detrimental effects on stigmatizing attitudes. Rather, together with a greater decrease in controllability beliefs, a greater increase in deterministic views of obesity contributed to the prediction of decreased stigmatizing attitudes four weeks post-intervention, with small effect size. This was true especially for women, who revealed in some previous investigations lower stigmatizing attitudes than men [12,14], and for individuals with high educational level.

## Conclusions

Based on two well-controlled, adequately powered studies, the newly developed brief, interactive intervention educating about gene × environment interactions in the etiology of obesity was found to be useful to decrease weight stigma, at least in the short term and in individuals with high educational level. In a comprehensive investigation of mechanisms of change and potential side-effects, the results further lended some support to the prediction of attribution theory [11], in that a focus on uncontrollable—genetic—factors (moderated by environmental influences that are at least partly controllable) can attenuate controllability beliefs (Study 1), which predicts lesser weight stigma (Study 2). Beyond fostering genetic causal attributions and knowledge, the intervention further led to an increase in beliefs in genetic determinism that was not found to be harmful, as previously cautioned [6,31,33], but contributed to decreased stigmatizing attitudes. Nevertheless, future research should rule-out any harmful effects of biogenetic causal information on individuals with obesity themselves. One study from the obesity field showed that providing genetic information did not have any effect on weight stigma internalization of individuals with overweight or obesity [47], a variable highly correlated with mental and physical health [48].

Limitations of this study include the use of different follow‐up periods (2 versus 4 weeks) and types of samples (student versus population sample), making the differences in results of Study 1 and Study 2 difficult to interpret. An assessment of the mechanisms of change at time points prior to the primary endpoint would be desirable in order to examine mediational effects. Related to the brevity of the intervention, it was not possible to discern effects of specific components. Further, stigmatizing attitudes may have been reduced via other mechanisms than studied here. For example, empathy has been found to be inversely associated with biogenetic attributions [49], and the intervention’s guided imagery task may have increased empathy). While this study addressed the development and initial evaluation of a new stigma reduction intervention, in future studies effects could be compared to other control conditions, for example, providing genetic information, or providing gene × environment information without other intervention components, in order to further dismantle intervention effects. Using a sample more representative of the population than the relatively young sample in Study 2 (mean population age 43.9 years) [50] would bolster generalizability of findings. Moreover, it would be desirable to examine effects on additional outcomes, including body image, self-efficacy, depression, eating behavior, or physical activity, and behavioral outcomes (e.g., social distance). Further, more work on the assessment of the belief in genetic determinism would be desirable, for example, clarifying its nature as a categorical or continuous concept [51].

Given that potent weight stigma reduction programs with sustainable success are widely lacking [5, 18], more research is warranted to develop efficacious interventions targeting both explicit and implicit stigmatizing attitudes in individuals from all educational backgrounds. Regarding the challenge to develop interventions to improve implicit stigmatizing attitudes [52], this can most likely be achieved by intensive multi-component interventions [27]. Other approaches that could be tested are, for example, evaluative conditioning or attentional bias modification [52]. Once safety and efficacy are further confirmed, interventions like the one described could be one low-cost and highly disseminable component, albeit with limited sustainability, in the societal challenge of reducing weight stigma. Because of the substantial individual and societal stigma-related burden [2,53], interventions to reduce weight stigma should become part of public health strategies, for example, within larger efforts towards health promotion or prevention of obesity and eating disorders.

## Supporting Information

S1 Data Study 1 Data. (SAV)

S2 Data Study 2 Data. (SAV)
